# Stress Cardiomyopathy Following Multivisceral Transplantation: A Case Report

**DOI:** 10.7759/cureus.78851

**Published:** 2025-02-11

**Authors:** Fouad G Souki, Nicolas Caram, Eslam Fouda, Ramona Nicolau-Raducu

**Affiliations:** 1 Anesthesiology, Jackson Memorial Hospital, University of Miami, Miami, USA

**Keywords:** ecmo, heart failure, intestine transplant, left ventricular systolic dysfunction, liver transplant, multiorgan transplant, multivisceral transplant, stress cardiomyopathy, takotsubo cardiomyopathy, venovenous bypass

## Abstract

Multivisceral transplantation (MVT) is an extraordinarily complex surgical procedure that imposes significant physiological stress driven by hemodynamic instability, fluid shifts, and severe bleeding. These factors can profoundly affect cardiovascular function and increase the risk of complications. Stress cardiomyopathy (Takotsubo syndrome), a transient non-ischemic heart failure characterized by acute left ventricular dysfunction, is a recognized complication of major surgeries but has not previously been reported in the context of MVT. This case report describes the occurrence, diagnosis, perioperative management, and favorable outcome of stress cardiomyopathy in a patient undergoing MVT with intraoperative venovenous extracorporeal membrane oxygenation support. It highlights the diagnostic criteria for stress cardiomyopathy, including echocardiographic findings and the application of the InterTAK score. It emphasizes the critical importance of heightened awareness, vigilance, and timely intervention to achieve a favorable clinical outcome.

## Introduction

Multivisceral transplantation (MVT), a highly complex surgical procedure involving en-bloc transplantation of the stomach, pancreas, intestine, and liver, is primarily indicated for patients with irreversible intestinal failure (e.g., short gut syndrome, dysmotility, tumors) or diffuse portomesenteric thrombosis [1]. Despite its clinical necessity, MVT remains a rare and high-risk operation, with fewer than 50 adult cases performed annually in the United States [1,2].

The procedure’s prolonged operative duration, coupled with significant hemodynamic instability, massive fluid shifts, and life-threatening thrombotic or hemorrhagic complications, imposes extraordinary stress on the cardiovascular system [3-5]. Advanced circulatory support strategies, such as venovenous bypass, are occasionally employed to mitigate hemodynamic fluctuations and optimize visceral perfusion [6].

Stress cardiomyopathy (Takotsubo syndrome), a transient form of acute heart failure triggered by physical or emotional stressors, is characterized by catecholamine-induced microvascular spasm, myocardial stunning, and reversible left ventricular (LV) systolic impairment [7,8]. While well-documented in liver transplantation (2% incidence, with over 200 cases reported since 2007) [9-15], its occurrence following MVT has not been previously described in the literature. Given the heightened physiological demands that surpass those of isolated liver transplantation, the risk of stress cardiomyopathy in this population is likely elevated, though unrecognized.

We present the first documented case of stress cardiomyopathy in a patient undergoing MVT with intraoperative venovenous extracorporeal membrane oxygenation (VV-ECMO) support. This report highlights the diagnostic challenges, perioperative management strategies, and critical role of multidisciplinary vigilance in detecting and managing this life-threatening complication to achieve a favorable outcome.

## Case presentation

Preoperative

A 61-year-old man with hepatitis C and alcoholic cirrhosis complicated by extensive mesenteric thrombosis presented from home for MVT. His medical history included esophageal varices treated with banding, diuretic-resistant ascites requiring weekly paracentesis due to extensive portal vein thrombosis, spontaneous bacterial peritonitis, and mesenteric thrombosis extending from the portosplenic confluence into the superior mesenteric vein, main portal vein, and splenic vein. Four attempts at intrahepatic portosystemic shunt placement via various approaches (jugular, splenic, femoral) had been unsuccessful more than three years ago. He was successfully treated for hepatitis C virus with sofosbuvir, velpatasvir, and voxilaprevir, achieving a sustained virologic response.

His medication regimen included bumetanide and spironolactone for ascites management, ciprofloxacin for infection prophylaxis, and hydrocodone/acetaminophen (7.5/325 mg) for pain control associated with hernias worsened by ascites.

The patient had no history of coronary artery disease, myocardial infarction, stroke, heart failure, or valvular heart disease. A dobutamine stress echocardiogram performed nine months earlier showed normal resting wall motion and no stress-induced electrocardiographic or echocardiographic evidence of ischemia or wall motion abnormalities, with a heart rate reaching 96% of the predicted maximum (155 beats per minute). A preoperative echocardiogram one month prior revealed normal LV systolic function with an ejection fraction (EF) of 55-60%, a left atrium volume index of 21.2 mL/m², and an E/e' ratio of 6.4, indicating normal LV end-diastolic pressure. Right ventricular systolic function was also normal, with a tricuspid annular plane systolic excursion (TAPSE) of 27 mm. A preoperative electrocardiogram (ECG) demonstrated sinus rhythm with premature atrial contractions and a QTc interval of 428 ms, consistent with his ECG findings from eight months earlier.

Laboratory evaluation revealed significant coagulopathy, including a prolonged prothrombin time of 22.7 seconds, an international normalized ratio of 1.93, and low fibrinogen levels of 137 mg/dL. Sodium was critically low at 125 mmol/L, potassium at 4.4 mmol/L, blood urea nitrogen at 35 mg/dL, and creatinine at 0.60 mg/dL. Additional findings included hypoalbuminemia (3.0 g/dL), elevated total bilirubin (2.4 mg/dL), anemia with a hemoglobin level of 9.2 g/dL, and a hematocrit of 28%. The platelet count was markedly decreased at 34 x 10³/μL. These findings resulted in a calculated Model for End-Stage Liver Disease-Sodium (MELD-Na) score of 26, indicating significant disease severity and a 40% six-month mortality risk.

Intraoperative

The procedure presented significant challenges due to the recipient's multiple varices, severe portal hypertension, extensive thrombosis in the main mesenteric vessels, and a small aneurysm in the infrarenal aorta. As is customary during MVT, the arterial inflow was established through an end-to-side anastomosis of the donor aortic segment to the recipient's aorta. Venous outflow was achieved by connecting the donor's inferior vena cava (IVC) to the recipient's IVC utilizing the piggyback technique. The donor's visceral block, comprising the liver, stomach, pancreaticoduodenal complex, small intestine, and a segment of the large intestine, was subsequently connected to the vascular orifices and successfully reperfused.

**Figure 1:**
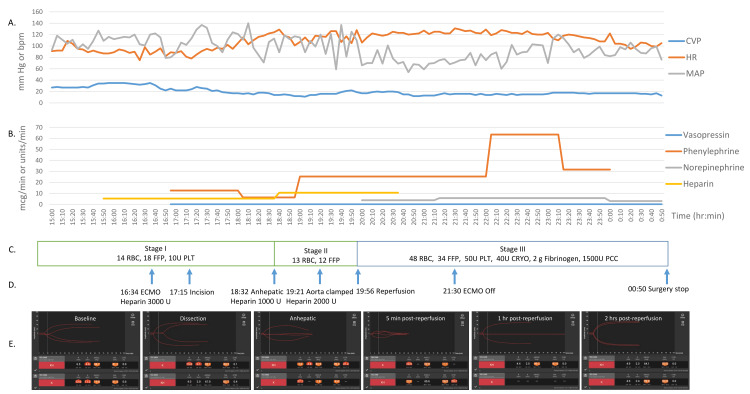
Timeline of intraoperative (A) vitals, (B) vasopressors, (C) blood products, (D) heparin administration, and (E) thromboelastogram CVP: central venous pressure, MAP: mean arterial pressure, HR: heart rate, RBC: packed red blood cell, FFP: fresh frozen plasma, PLT: platelets, U: units, ECMO: extracorporeal membrane oxygenation, CRYO: cryoprecipitate, PCC: prothrombin complex concentrate, hr: hour, min: minutes, bpm: beats per minute.

Anesthesia was initiated with rapid sequence induction using propofol and succinylcholine, followed by maintenance with sevoflurane, fentanyl, and continuous cisatracurium infusion. Vascular access was established via a 9 Fr double-lumen introducer and a 14 Fr triple-lumen catheter in the left internal jugular vein, complemented by brachial and radial arterial lines. Hemodynamic monitoring included transesophageal echocardiography (TEE) and FloTrac®/Vigileo to measure cardiac output and systemic vascular resistance. Vasopressors, including phenylephrine (10-80 mcg/min), norepinephrine (1-5 mcg/min), and vasopressin (0.04-0.08 units/min), were titrated to maintain a mean arterial pressure (MAP) > 65 mm Hg during critical phases, such as tissue dissection, bleeding, and IVC/aorta clamping and unclamping (Figure 1A-1B). During reperfusion, the patient received a 100 mcg epinephrine bolus, followed by two additional 30 mcg doses a few minutes apart. Post-reperfusion vasopressor requirements temporarily increased but subsided by the end of the surgery. Additional treatment for vasoplegia, such as methylene blue or hydroxocobalamin, was not required as blood pressure was effectively maintained with moderate vasopressor infusions. Aside from sinus tachycardia, no intraoperative ECG or echocardiographic abnormalities were observed.

Venovenous ECMO was initiated by a cardiothoracic surgeon using a 20 Fr catheter in the right internal jugular vein and a 25 Fr catheter in the left femoral vein, placed under fluoroscopic and TEE guidance. ECMO provided venovenous bypass with a flow rate of 3 L/min, ensuring hemodynamic stability during organ manipulation and IVC cross-clamping. Decannulation was performed following reperfusion once venovenous ECMO support was no longer necessary (Figure 1D).

Baseline thromboelastography (TEG) revealed a prolonged reaction time (R) of 12 minutes, a prolonged K of 11.2 minutes, a reduced alpha angle of 19.6°, and a decreased maximum amplitude of 31.5 mm (Figure 1E). Per our protocol, a continuous intravenous heparin infusion at 500 units/hour was initiated at incision to prevent intracardiac thrombus formation and discontinued after reperfusion. Additional heparin boluses were administered as needed, including 3000 units during venovenous ECMO initiation, 1000 units at the onset of the anhepatic phase, and 2000 units during aortic partial clamping, guided by TEG results.

Blood product transfusion was guided by estimated blood loss and arterial blood gas analysis to maintain a hematocrit above 25%, using a 1:1 ratio of packed red blood cells (RBC) to fresh frozen plasma (FFP). The patient required extensive blood product support, including 22 units of washed RBC to decrease potassium load, 53 units of unwashed RBC, 9 units of cell-saver RBC, 64 units of FFP, 60 units of platelets, and 40 units of cryoprecipitate (Figure 1C).

To reduce the risk of excessive clotting and intracardiac thrombus formation, platelets, cryoprecipitate, and coagulation factors (e.g., fibrinogen 2 grams, prothrombin complex concentrate 1500 units) were administered post-reperfusion to treat coagulopathy guided by TEG. Fibrinolysis and the residual heparin effect identified on TEG were managed with a 1 g bolus of aminocaproic acid and 10 mg of protamine post-reperfusion (Figure 1C, 1E).

Additional intraoperative fluid support included 7 liters (L) of Isolyte® and 1.5 L of 5% albumin. Intravenous calcium chloride (7 g) and sodium bicarbonate (700 mEq) were administered to treat hypocalcemia and metabolic acidosis associated with significant bleeding and massive transfusion. The estimated blood loss was 24 L.

The MVT procedure lasted eight hours and 28 minutes, and the cold ischemia time was five hours. Intraoperative analgesia included 250 mcg of fentanyl and 2 mg of hydromorphone. Immunosuppression was initiated with 500 mg methylprednisolone.

Postoperatively, the patient was transferred to the intensive care unit (ICU) intubated and requiring inotropic support with phenylephrine, norepinephrine, and vasopressin to maintain blood pressure at 118/84 mmHg with a heart rate of 102 beats per minute.

Postoperative

The patient initially demonstrated a favorable postoperative course, evidenced by decreasing vasopressor requirements and successful extubation 32 hours after surgery. However, on postoperative day (POD) 2, the clinical condition deteriorated with the onset of atrial fibrillation (AF), resulting in oxygen desaturation and necessitating reintubation. Management included starting an amiodarone infusion and increasing vasopressor support, after which the cardiac rhythm returned to the sinus.

**Figure 2:**
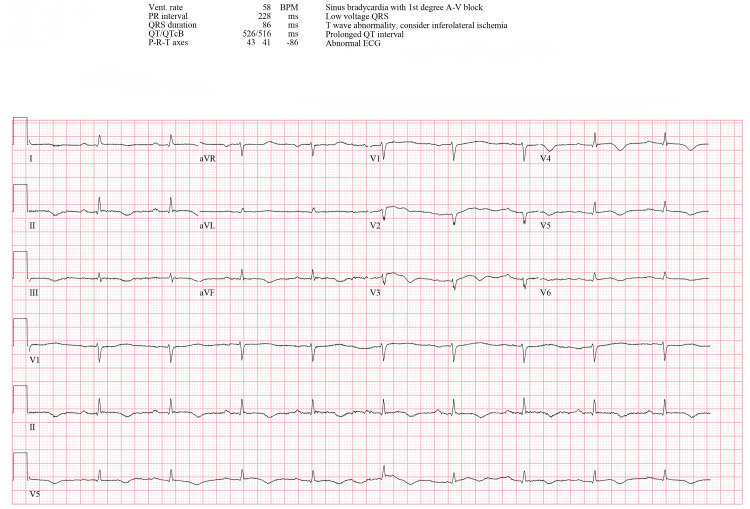
ECG showing first-degree atrioventricular block after AF treatment ECG: electrocardiogram, AF: atrial fibrillation, Vent. rate: ventricular rate, BPM: beats per minute, PR interval: PR interval (time between atrial and ventricular depolarization), QRS duration: QRS complex duration (ventricular depolarization time), QT/QTcB: QT interval/corrected QT interval (Bazett’s formula), P-R-T axes: P wave, R wave, and T wave axes, A-V block: atrioventricular block, QRS: QRS complex (ventricular depolarization wave)

**Figure 3:**
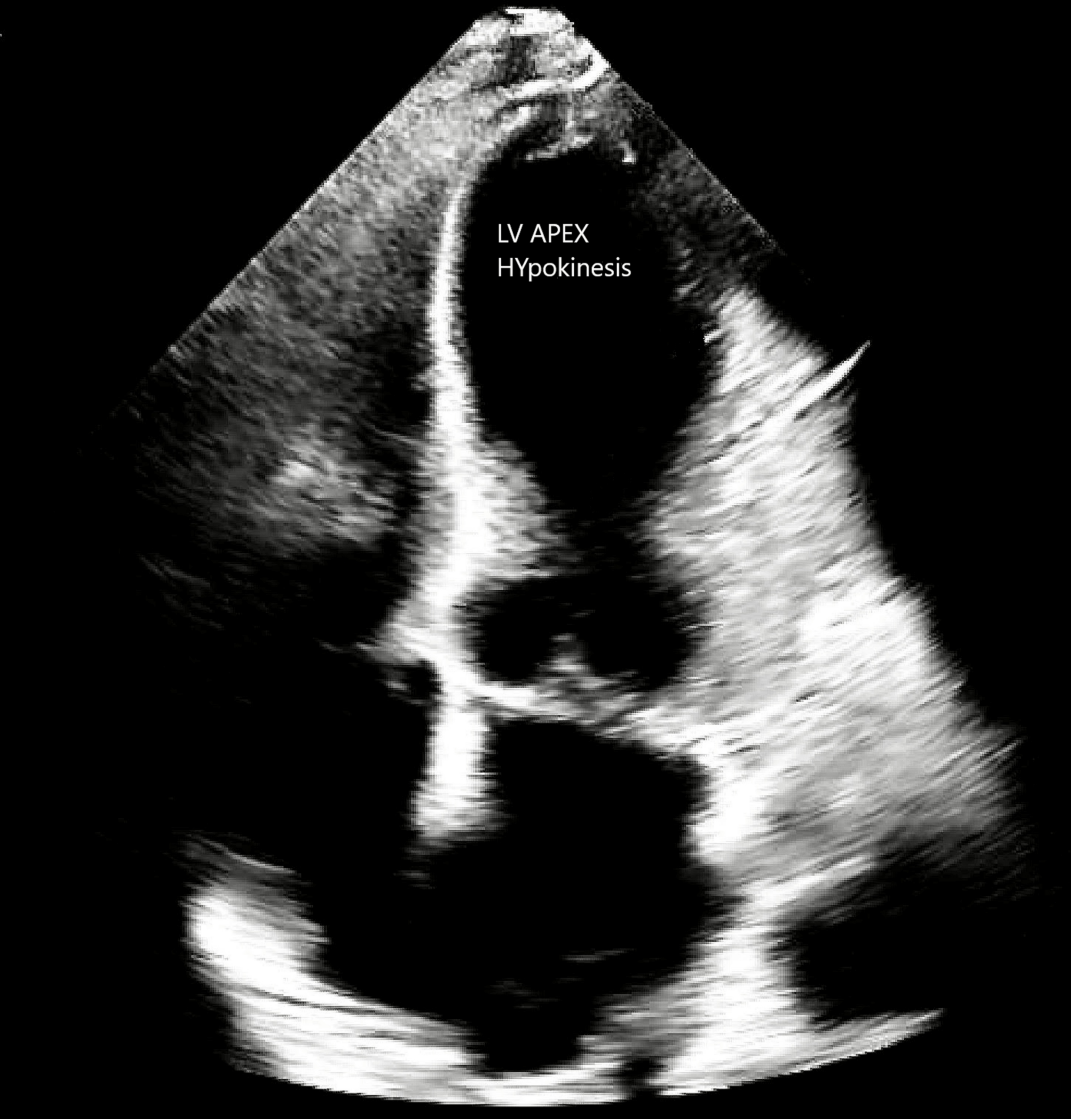
Transthoracic echocardiogram showing severe hypokinesis of the mid and apical segments. Abnormal LV wall motion pattern showing stress cardiomyopathy, apical variant LV: left ventricular

Without electrolyte abnormalities, the ECG revealed sinus bradycardia with first-degree atrioventricular block, low QRS voltage, inferolateral T-wave changes, and a prolonged QTc interval of 512 ms (Figure 2). Echocardiography showed an EF of 35-40%, along with severe left LV hypokinesis affecting the mid and apical segments of both the anterior and inferior walls (Figure 3). These findings were consistent with stress cardiomyopathy, specifically the apical variant.

**Figure 4:**
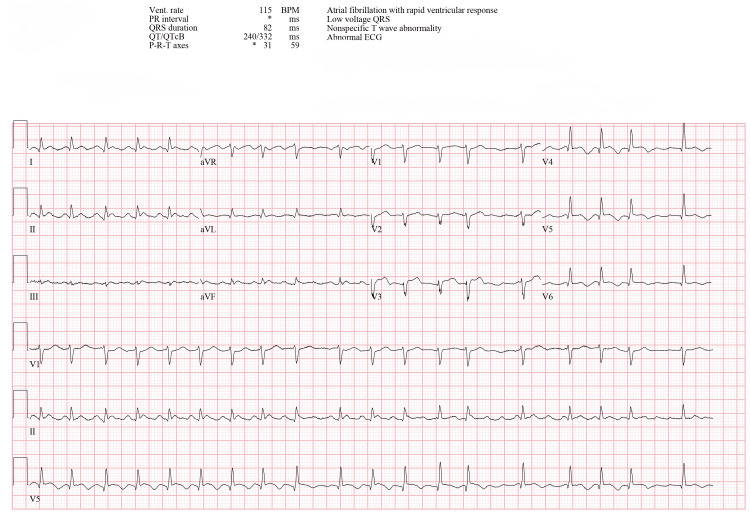
ECG showing AF POD 4 ECG: electrocardiogram, AF: atrial fibrillation, POD: postoperative day, Vent. rate: ventricular rate, BPM: beats per minute, PR interval: PR interval (time between atrial and ventricular depolarization), QRS duration: QRS complex duration (ventricular depolarization time), QT/QTcB: QT interval/corrected QT interval (Bazett’s formula), P-R-T axes: P wave, R wave, and T wave axes, A-V block: atrioventricular block, QRS: QRS complex (ventricular depolarization wave)

On POD 3, the patient underwent abdominal washout and closure, requiring increased vasopressor support and transfusion of 4 units of RBC along with 10 units of platelets. Laboratory results showed notably elevated troponin I at 2.510 ng/mL, B-type natriuretic peptide (BNP) at 10,700 pg/mL, and worsening renal function with a blood urea nitrogen of 44 mg/dL and creatinine of 1.4 mg/dL. On POD 4, AF recurred, and the patient remained completely off vasopressors (Figure 4).

On POD 5, metoprolol was initiated for rate control in persistent AF. Pharmacologic cardioversion remained challenging; however, the patient maintained stable hemodynamics without requiring vasopressors. By POD 9, the cardiac rhythm converted to normal sinus rhythm, although there was poor R-wave progression and persistent T-wave abnormalities with QTc prolongation. On POD 11, repeat echocardiography demonstrated a moderately reduced LV systolic function with an EF of 35-40% and a TAPSE of 20 mm, still suggestive of stress cardiomyopathy. The patient was successfully extubated on this day. AF recurred on POD 15 and spontaneously resolved on POD 18, with ECG showing sinus rhythm at 89 beats per minute and premature atrial complexes. A subsequent echocardiogram on POD 23 revealed normalization of LV systolic function, with an EF of 55-60%. The ECG then showed sinus rhythm, premature atrial complexes, lateral T-wave abnormalities, and a persistently prolonged QT interval at 492 ms. On POD 24, the patient was transferred to the general ward for activities of daily living and functional mobility rehabilitation.

## Discussion

This case represents the first reported occurrence of stress cardiomyopathy in an MVT recipient, underscoring the profound physiological strain inherent to this procedure. MVT-encompassing en-bloc transplantation of the stomach, pancreas, intestine, and liver entails prolonged ischemia-reperfusion cycles, multi-visceral vascular dissection, and simultaneous reperfusion of multiple organs. These factors amplify systemic inflammation, hemodynamic instability, and catecholamine surges, creating a “perfect storm” of myocardial stressors [7,8]. The patient’s clinical course illustrates how MVT-specific challenges, including prolonged operative duration (>8 hours), massive hemorrhage (24 L), transfusion requirements (84 units RBC), and vasopressor dependence (norepinephrine, vasopressin, phenylephrine, epinephrine), precipitated stress cardiomyopathy despite advanced intraoperative support with VV-ECMO. Stress cardiomyopathy manifested on POD 2, with a resolution by POD 23, aligning with the typical postoperative onset (one to five days) and recovery timeline (≤4 weeks) [12].

Stress cardiomyopathy is defined by transient LV systolic dysfunction not attributable to coronary artery disease, pheochromocytoma, or myocarditis, along with new electrocardiographic abnormalities or modest troponin elevation. The myocardial dysfunction typically extends beyond an epicardial vascular distribution [16,17]. Diagnosis in this case was established using echocardiography, which identified hallmark features such as apical ballooning and hypokinesis. Laboratory findings included elevated troponin I (peak: 2.5 ng/mL) and BNP (peak: 14,300 pg/mL), alongside ECG changes like QTc prolongation and ST-segment deviations all serially tracked during evaluation [16]. Troponin elevation in this condition is attributed to catecholamine-mediated myocardial stunning and microvascular dysfunction, while BNP rise correlates with ventricular wall stress and diastolic impairment [7]. The InterTAK diagnostic score (55 points), designed to differentiate acute coronary syndrome from stress cardiomyopathy, strongly supported the diagnosis, with points allocated for physical (surgical) and emotional triggers, QTc prolongation, and the absence of ST depression [18]. TAPSE, a key echocardiographic measure of right ventricular function, remained normal, excluding significant right ventricular involvement, a finding consistent with typical apical-predominant stress cardiomyopathy variants [7].

Arrhythmias, particularly AF, are commonly seen in liver transplant recipients with stress cardiomyopathy [9]. Determining whether AF is the cause or result of cardiomyopathy can be difficult. In this case, the LV dysfunction due to stress cardiomyopathy likely triggered AF rather than the reverse. Paroxysmal AF (on POD 2, 4-9, and 15-18) likely developed from catecholamine-induced atrial strain and systemic inflammation, both worsened by LV dysfunction associated with stress cardiomyopathy. The reduced atrial contribution to cardiac output intensified hemodynamic instability, creating a vicious cycle where tachycardia predisposed the patient to AF, and AF, in turn, placed additional strain on the LV. Early rhythm or rate control with amiodarone and metoprolol was crucial in breaking this cycle and preventing further myocardial stress [9].

Tachycardia-induced cardiomyopathy (TIC) typically arises from sustained tachyarrhythmias (such as AF, flutter, or supraventricular tachycardia) over a period of weeks to years, resulting in myocardial remodeling and global left LV dysfunction. Recovery from TIC generally requires several weeks to months of rhythm or rate control [19]. As a result, TIC was ruled out, as paroxysmal AF occurred simultaneously with, rather than preceding, LV dysfunction; myocardial impairment was not global; and the EF recovery within 23 days was inconsistent with TIC's typical longer recovery time [19].

Several factors have been associated with an increased risk of stress cardiomyopathy in liver transplant recipients, many of which may also apply to MVT. Preoperatively, demographic factors such as older age, Caucasian ethnicity, female sex, and a history of alcoholic cirrhosis have been shown to increase susceptibility [9]. Pretransplant laboratory abnormalities, particularly a high MELD score (>27), hyponatremia, elevated BNP, and anemia (Hb <8 g/dL), have also been implicated [12,13,16,20,21]. Additionally, underlying cardiac conditions, including arrhythmias, a history of heart failure, and echocardiographic findings such as cirrhotic cardiomyopathy, wall motion abnormalities, diastolic dysfunction, prolonged QTc interval, impaired global longitudinal strain, and increased left atrial volume index, further elevate the risk of stress cardiomyopathy [20-25]. Intraoperatively, excessive catecholamine administration, the use of vasopressors (e.g., norepinephrine or epinephrine), inadequate anesthetic depth, and insufficient opioid administration have been identified as potential triggers [12,13]. Furthermore, significant blood transfusion (>11 units RBC) and excessive fluid administration can exacerbate the risk [12,13].

Our patient exhibited multiple stress cardiomyopathy risk factors common in liver transplant cohorts, including alcoholic cirrhosis, a MELD score of 26, hyponatremia (Na 125 mmol/L), intraoperative catecholamine use (epinephrine, norepinephrine), massive hemorrhage (24 L), extensive transfusions (84 units RBC), and substantial fluid administration. While non-modifiable factors (age, MELD score) increased, baseline susceptibility and modifiable intraoperative stressors, aggressive fluid resuscitation, cumulative epinephrine dosing, and vasopressor reliance, likely amplified myocardial injury. The preoperative cardiac risk was limited to premature atrial contractions, with no evidence of cirrhotic cardiomyopathy or diastolic dysfunction.

VV-ECMO at a flow rate of 3 L/min was utilized to maintain hemodynamic stability by diverting venous blood from the IVC and portal circulation and preserving cardiac preload. This strategy has been shown to reduce intraoperative blood loss, shorten the anhepatic phase, and lower the risk of postoperative respiratory complications. By preserving stable hemodynamics and mitigating myocardial strain, VV-ECMO helps prevent ischemia-reperfusion injury and post-reperfusion syndrome, thereby reducing vasopressor requirements and myocardial injury [6]. We routinely use VV-ECMO during MVT, although the cannulation and decannulation process adds approximately one hour to the operative time. Despite its benefits, VV-ECMO could not prevent stress cardiomyopathy, emphasizing the need for adjunct strategies to address multifactorial myocardial stressors.

Post-transplant stress cardiomyopathy has been associated with worse outcomes, including AF, venous thrombosis, pulmonary embolism, stroke, delayed extubation, prolonged ICU and hospital stays, and increased mortality [13,20,21,23,24]. In our patient, stress cardiomyopathy contributed to AF, reintubation, and extended ICU hospitalization.

Management of stress cardiomyopathy is largely supportive, focusing on hemodynamic stabilization and addressing underlying triggers [26]. Key measures include fluid resuscitation, cautious use of vasoactive agents, correction of electrolyte imbalances, and managing complications such as cardiogenic shock, LV outflow tract obstruction, arrhythmias, or thrombosis. While inotropes may be required in severe cases, recent evidence suggests they may worsen outcomes, favoring non-catecholaminergic agents like vasopressin and milrinone [26-28]. For refractory cases, mechanical circulatory support, including venoarterial ECMO or intra-aortic balloon pump, may be necessary to reduce vasoactive medication requirements and improve outcomes [29-32]. Early diagnosis enabled tailored cardiovascular management in our patient, prioritizing judicious fluid resuscitation, avoiding additional catecholamines, and rate control for concomitant AF.

## Conclusions

This case highlighted the complexity of MVT and the potential for stress cardiomyopathy as a serious complication. Key lessons include the need for heightened awareness, perioperative vigilance, early diagnosis with echocardiography, biomarker monitoring (troponin I, BNP), and tailored cardiovascular care to achieve a favorable outcome. Management strategies include minimizing modifiable stressors such as judicious fluid resuscitation, balanced vasopressor use, and aggressive arrhythmia control to mitigate myocardial injury. While intraoperative VV-ECMO aids in hemodynamic stability, its limitations underscore the need for adjunct approaches to address multifactorial myocardial stressors. Future research must delineate the incidence, refine risk stratification, and explore preventive strategies to mitigate this life-threatening complication. We hope sharing this experience can enhance understanding and preparedness among transplant teams and improve outcomes.
